# Metabolism of Cryptic Peptides Derived from Neuropeptide FF Precursors: The Involvement of Insulin-Degrading Enzyme

**DOI:** 10.3390/ijms150916787

**Published:** 2014-09-22

**Authors:** Giuseppe Grasso, Przemyslaw Mielczarek, Magdalena Niedziolka, Jerzy Silberring

**Affiliations:** 1 Department of Chemical Sciences, University of Catania, Viale Andrea Doria 6, 95125 Catania, Italy; 2 Department of Biochemistry and Neurobiology, AGH University of Science and Technology, Mickiewicza 30, 30-059 Krakow, Poland; E-Mails: przemyslaw.mielczarek@agh.edu.pl (P.M.); mwasielak@googlemail.com (M.N.); 3 Centre for Polymer and Carbon Materials, Polish Academy of Sciences, Sklodowskiej-Curie 34, 41-819 Zabrze, Poland; E-Mail: jerzy.silberring@agh.edu.pl

**Keywords:** IDE, neuropeptide, metalloprotease, cryptome, mass spectrometry, surface plasmon resonance

## Abstract

The term “cryptome” refers to the subset of cryptic peptides with bioactivities that are often unpredictable and very different from the parent protein. These cryptic peptides are generated by proteolytic cleavage of proteases, whose identification *in vivo* can be very challenging. In this work, we show that insulin-degrading enzyme (IDE) is able to degrade specific amino acid sequences present in the neuropeptide pro-NPFF_A_ (NPFF precursor), generating some cryptic peptides that are also observed after incubation with rat brain cortex homogenate. The reported experimental findings support the increasingly accredited hypothesis, according to which, due to its wide substrate selectivity, IDE is involved in a wide variety of physiopathological processes.

## 1. Introduction

The terms “cryptic peptides” and “cryptome” have been used for a few decades to indicate the pools of peptides that are formed by the proteolytic action of peptidases on precursor proteins. In many cases, it has been demonstrated that such peptides have biological properties and activities that are distinct from their precursor proteins [1,2] and, for this reason, very recently, the investigation of the biochemical mechanisms, through which they are generated, has been pursued by many researchers [3,4]. The word “cryptomics” has also been introduced to describe the systemic approach of finding crypteins *in vitro* [5]. In this scenario, cryptic peptides, which are normally present in the brain, *i.e.*, neuropeptides (NPs), seem to have very important modulatory roles in controlling our mood, energy levels, pain and pleasure perception, *etc.* [6]. NPs are considered the largest class of messengers in the central nervous system (CNS), and as other cryptic peptides, their formation and homeostasis are tightly regulated by the action of various proteases acting on the precursor proteins from which they are generated [7]. Particularly, NPs released from the NPFF precursor (pro-NPFF_A_) have been recently reported to be involved in several processes, such as pain transmission [8], the action of drugs of abuse [9], cardiovascular functions, appetite, thirst and body temperature [10]. Very recently, we have reported the discovery of a novel amino acid sequence derived from pro-NPFF_A_, NAWGPWSKEQLSPQA, named NPNA, from neuropeptide (NP) and its flanking amino acids (N and A), which is able to block the expression of conditioned place preference induced by morphine and reverses the antinociceptive activity of morphine in the tail-immersion test in rats [11]. This peptide, in contrast to the previously described fragments derived from pro-NPFF_A_ (*i.e.*, NPFF: FLFQPQRF-NH_2_; NPAF: AGEGLSSPFWSLAAPQRF-NH_2_; NPSF: SLAAPQRF-NH_2_), does not have the PQRF-amidated sequence at the *C*-terminus [12]. Moreover, exogenous NPNA (residues 85–99 in pro-NPFF_A_) [11], apart from having NPFF-like behavioral activity in rats [11], also modulates the activity of genes coding for three α subunits of the G protein in three different brain regions [13]. However, the exact processes by which NPNA and other NPs derived from pro-NPFF_A_ are formed remain unclear. Because of the wide variety of proteases present in the brain, the understanding of the proteolytic processes, which are responsible for the formation and homeostasis of these NPs, represents a very important and challenging task.

Insulin-degrading enzyme (IDE) is a zinc metalloprotease that has recently attracted the interest of the scientific community because of its broad specificity for peptide substrates [14,15,16,17] and its ubiquitous localization [18]. Indeed, both factors make this enzyme potentially available for a variety of biomolecular processes involved in homeostatic and pathological conditions, such as Alzheimer’s disease (AD). In experiments using IDE-deficient mice, Aβ catabolism is considerably reduced with an increase of endogenous Aβ [19], and IDE degrades Aβ in conditioned medium from neuronal cultures [20]. Moreover, the transgenic overexpression of IDE results in lowered Aβ levels and reduced or completely prevented the plaque formation observed in AD [21]. More recently, it has been reported that IDE, apart from regulating insulin [22] and Aβ peptides levels [23], is overexpressed *in vivo* in tumors of the CNS, and a novel role for IDE as a heat shock protein has also been proposed [24].

In this work, we wanted to provide insight into the proteolytic processes regulating the metabolism of some of the abovementioned NPs derived from pro-NPFF_A_. For this purpose, we have applied electrospray ionization mass spectrometry (ESI-MS) for the analysis of the NPs generated during the incubation of various precursor NPs in brain cortex homogenates. We have then investigated the cryptic peptides that are generated by the digestion with IDE of the same bioactive NPs derived from pro-NPFF_A_. The experimental findings indicate that IDE is involved in the metabolism of NPs derived from pro-NPFF_A_.

## 2. Results and Discussion

**Figure 1:**
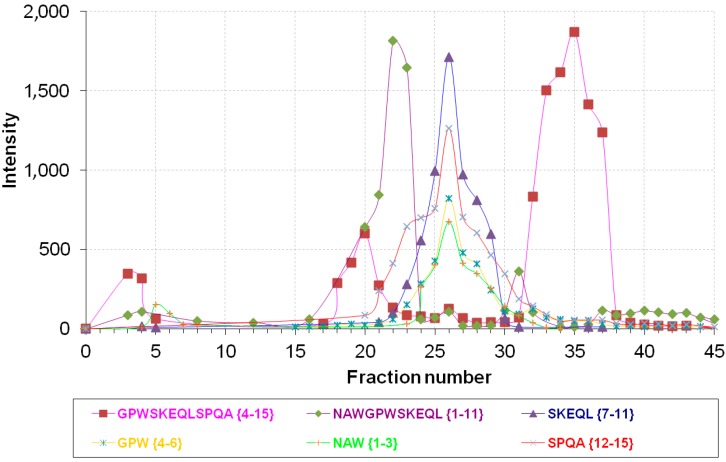
Intensity distribution of major products of NPNA (NAWGPWSKEQLSPQA {1–15}) metabolism in Fractions 1–45 from rat brain cortex separation on an Econo-Pac Q column.

**Figure 2:**
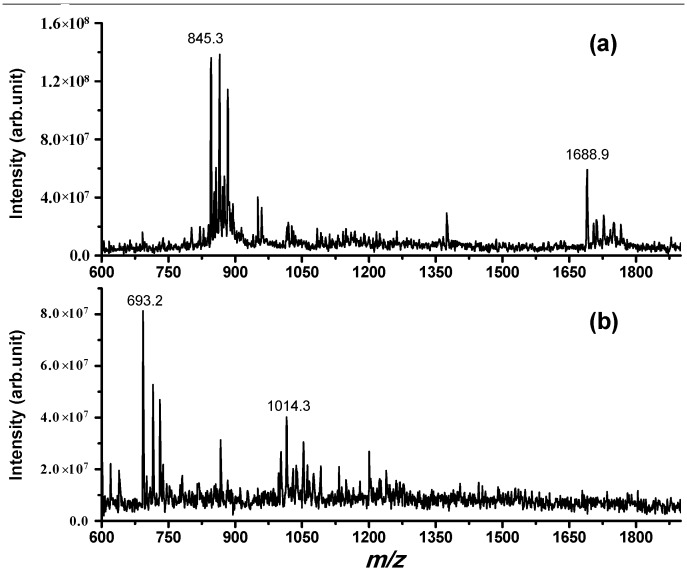
Electrospray ionization (ESI) mass spectrum obtained for a PBS solution incubated for 2 h at 37 °C containing (**a**) (NPSA 1–15) = 50 μM; (**b**) (NPSA 1–15) = 50 μM and (IDE) = 8 nM; while in (**a**), only the full-length peptide is detected (with the addition of H^+^, Na^+^ and K^+^); in (**b**), the two main fragments derived from cleavage site Trp6–Ser7 are detected, confirming the proteolytic action of IDE on this peptide.

Firstly, we wanted to elucidate the possible mechanisms responsible for NPNA proteolysis. The investigation of this process, consisting of intracellular degradation of peptides by enzymes present in brain cortex, enabled us to confirm the biological activity of the tested compounds in analgesia and drug dependence [13]. For this purpose, proteases metabolizing NPNA in rat brain cortex were applied. Products of proteolysis were identified using ESI-MS and are summarized in Table 1. Based on the integration of the peak area of newly-created peptides, only three fractions, with the highest product intensity, were taken into account for further time course metabolic process (Figure 1). The highest product intensity was observed after 4 h cleavage (the time-dependent data are reported in Figure S1). The activity of enzymes is not specific: proteases cleave peptides mainly at the carboxyl side of tryptophan and leucine [25]. Therefore, these results suggest the existence of several peptidases converting NPNA peptide to shorter bioactive fragments responsible for the analgesic activity of this rat cryptein. In this case, we have confirmed that IDE is not responsible for NPNA degradation, because incubation with IDE does not produce any NPNA fragments at all incubation times investigated (up to 24 h, the mass spectrum was the same as the one recorded without IDE; data not shown). One could argue that the absence of degradation could be attributed to the short length of the peptide, because, with some exceptions, most IDE substrates are considerably longer than 15 amino acids [16]. This common feature among IDE substrates has been explained by considering that IDE has an exosite that is an evolutionary-conserved substrate-binding site 30 Å away from the catalytic groove and serves as an anchor to attach the *N*-terminal end of IDE substrates [26]. In order to assess if the absence of proteolytic fragments is due to the short length of the substrate or to the lack of sequence specificity, we have carried out the same proteolytic experiment using another NP, named NPSA (SAWGSWSKEQLNPQA {1–15}), which has the same number of amino acids as NPNA, but contains three substitutions at Positions 1, 5 and 12 and is obtained from mouse pro-NPFF_A_ [12]. As is reported in Figure 2, one main cleavage site is observed in this case (between Trp6 and Ser7; fragments at *m*/*z* 693.2 and 1014.3 corresponding, respectively, to SAWGSW and SKEQLNPQA were confirmed by MS/MS experiments; see Figure S2). Degradation of NPSA by IDE is time-dependent and is completed within two hours for a solution that has (IDE) = 1/6000 (NPSA), as is confirmed by the disappearance of the NPSA molecular peak from the mass spectrum (Figure 2b). These results demonstrate that IDE is able to degrade peptides as short as 15 amino acids in a sequence-specific manner.

**Table 1:** Products of NPNA (NAWGPWSKEQLSPQA {1–15}) metabolism in major fractions from rat brain cortex separation. The most intense products on the mass spectrum (not shown) are marked in bold.

Fraction No.	Peptide Fragment	Sequence	*m*/*z* Value
23	**{1–11}**	**NAWGPWSKEQL**	**658.5 (2+)**
	{12–15}	SPQA	402.4
26	{1–11}	NAWGPWSKEQL	658.5 (2+)
	{1–3}	NAW	390.4
	{4–6}	GPW	359.4
	**{7–11}**	**SKEQL**	**604.6**
	{12–15}	SPQA	402.4
35	**{4–15}**	**GPWSKEQLSPQA**	**664.5 (2+)**

**Figure 3:**
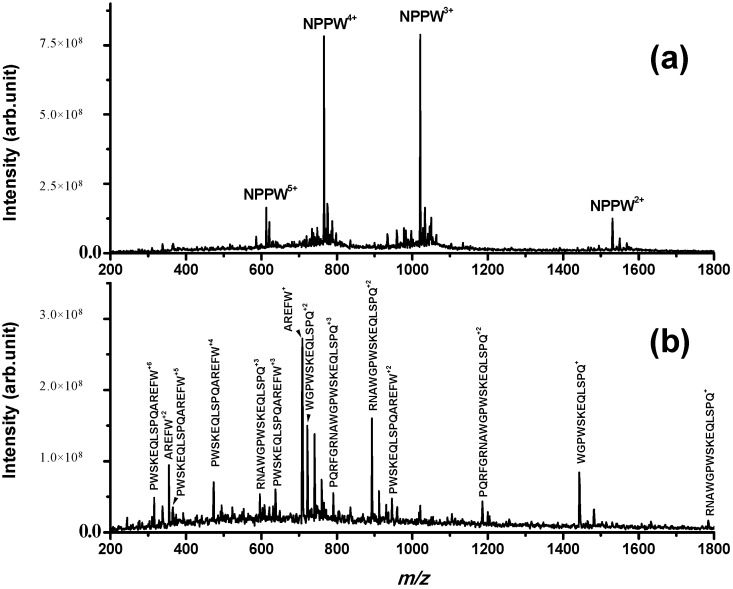
ESI mass spectrum obtained for a PBS solution incubated for 8 h at 37 °C containing (**a**) (NPPW 1–25) = 50 μM; (**b**) (NPPW 1–25) = 50 μM and (IDE) = 8 nM; while in (**a**), only the full-length peptide is detected; in (**b**), the fragments derived from cleavage sites Gly5–Arg6, Ala8–Trp9, Gly10–Pro11 and Gln20–Ala21 are detected, confirming the proteolytic action of IDE on this peptide. Other peaks assigned to the same fragments with the addition of Na^+^ and K^+^ are not labelled for simplicity.

However, even if IDE is not able to degrade NPNA, we have investigated if it is able to produce NPNA or some other peptides from longer pro-NPFF_A_ fragments. For this purpose, a new peptide sequence (PQRFGRNAWGPWSKEQLSPQAREFW) derived from rat pro-NPFF_A_ was tested as a possible IDE substrate. PQRFGRNAWGPWSKEQLSPQAREFW is an elongated form of NPNA and is named NPPW 1–25 from now on. NPPW 1–25 was incubated with IDE, and the results are reported in Figure 3. It can be observed that IDE is able to produce several fragments derived from cleavage sites Gly5–Arg6, Ala8-Trp9, Gly10-Pro11 and Gln20-Ala21. The detected fragments confirm the proteolytic action of IDE on NPPW 1–25 (fragments were confirmed by MS/MS experiments; as an example, see the fragmentation of peak at *m*/*z* 1442.7 assigned to fragment WGPWSKEQLSPQ in Figure S3). It is important to highlight that the major cleavage site is Gln20–Ala21, as the only peptide fragment that is not truncated at Gln20 (PWSKEQLSPQAREFW, that is NPPW 11–25) is generated only at longer incubation times (more than 2 h; data not shown). Therefore, in our experimental conditions, some peptide fragments different from NPNA were produced by the action of IDE on NPPW 1–25.

**Figure 4:**
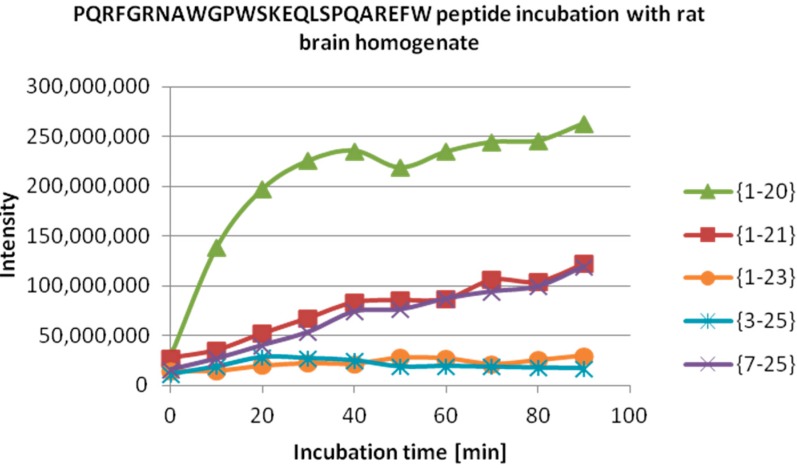
ESI-MS quantitative detection of peptide fragments produced by the degradation of (NPPW 1–25) in rat brain homogenate (see Table 2) at different incubation times. Note that the most abundant peptide fragment is NPPW 1–20. The same result was obtained with and without the addition of amastatin.

We then tested the degradation of NPPW 1–25 peptide after incubation with rat brain homogenate, with and without the addition of amastatin. The latter was added to exclude the activity of aminopeptidases that are abundant in CNS. Such activity is responsible, among others, for the inactivation of opioid peptides, by truncation of the *N*-terminal Tyr, thus preventing binding to opioid receptors. Indeed, the same results were recorded with and without this inhibitor. In Table 2, the assignment of the peptide fragments detected is reported, while in Figure 4, a quantitative detection of their amounts at different incubation times is also shown. Notably, the most abundant peak is NPPW 1–20, which indicates Gln20–Ala21 as the major cleavage site. Therefore, this result suggests that one of the proteases responsible for the degradation of NPPW 1–25 is very likely to be IDE, as the above reported *in vitro* experiments show that this enzyme is able to cleave the peptide at the same cleavage site, producing fragment PQRFGRNAWGPWSKEQLSPQ at high abundance, and is not inhibited by amastatin. A further proof of this thesis could be obtained by applying selective IDE inhibitors that could become more easily available in the near future [27].

**Table 2:** Products of NPPW 1–25 in major fractions from rat brain cortex separation. The most intense fragment on the mass spectrum is NPPW 1–20 (see Figure 4).

Peptide	Mass (Da)	Ion *m*/*z*	Charge	Sequence
{1–20}	2368.2	790.4	3^+^	PQRFGRNAWGPWSKEQLSPQ
{1–21}	2439.2	814.1	3^+^	PQRFGRNAWGPWSKEQLSPQA
{1–23}	2724.4	909.1	3^+^	PQRFGRNAWGPWSKEQLSPQARE
{3–25}	2832.4	945.1	3^+^	RFGRNAWGPWSKEQLSPQAREFW
{7–25}	2316.1	773.1	3^+^	NAWGPWSKEQLSPQAREFW

**Figure 5:**
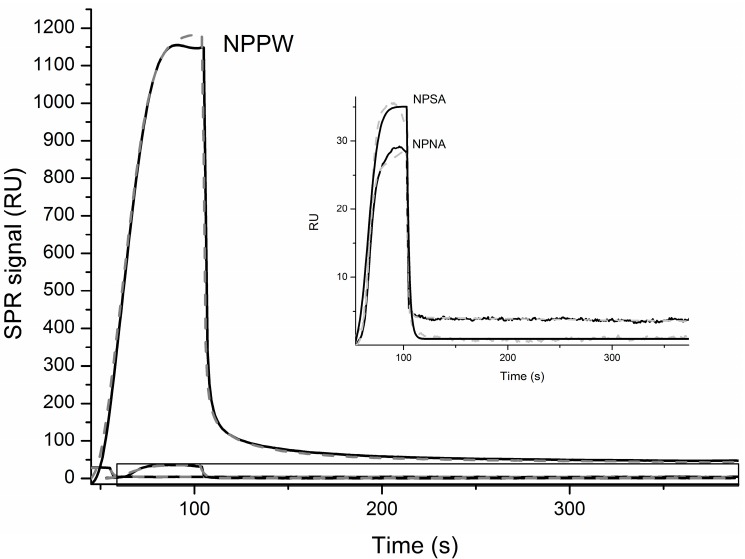
SPR curves obtained for the OneStep interaction of immobilized IDE and the indicated NPs. The inset is a zoom of the boxed area. Black lines are experimental data, while grey dashed lines are simulations obtained as discussed in the text.

Finally, in order to further characterize the interaction between IDE and the NPs reported above, we have immobilized IDE, as described in the Materials and Methods, and measured its affinity with the different NPs by Surface Plasmon Resonance (SPR). In Figure 5, the OneStep binding curves together with the simulations curves that produced the kinetic parameters reported in Table 3 are shown. It is important to highlight that, in order to fit the experimental curves, the two binding site interaction model was necessary in the case of NPSA and NPPW peptides. Particularly, as is shown in Table 3, the *K*_D_ values obtained are within the same order of magnitude of other IDE substrates [15,28]. The NPPW 1–25 peptide has the lowest *K*_D_ values for both binding sites, confirming a higher affinity of IDE toward this elongated sequence. The above reported MS results show that NPNA is the only peptide among the three investigated that is not cleaved by IDE and, interestingly, is also the only one whose SPR OneStep curve could be better fitted by the one binding site model. Therefore, it is easy to speculate that, in order to be an IDE substrate, a peptide has to bind to the enzyme by two binding sites, as has already been proposed by other groups [26]. However, such speculation needs to be further confirmed by applying the same SPR experimental procedure using many other different substrates.

**Table 3:** Kinetic parameters obtained by SPR experiments for the NPs indicated. Res SD values refer to the residuals.

Peptide	*k*_on1_	*k*_off1_	*K*_D1_	*k*_on2_	*k*_off2_	*K*_D2_	Res SD
NPNA	139.7	0.5562	3.98161 mM	–	–	–	2.578
NPSA	18.66	0.6637	35.55 mM	2.476	0.614	247.97 μM	3.521
NPPW	3180	0.354	111 μM	19	0.032	170 μM	0.647

## 3. Materials and Methods

### 3.1. Peptide Synthesis

NPNA was synthesized by the standard Fmoc-solid phase peptide synthesis method and purified by HPLC, using a preparative reverse-phase C18 column (Hypersil Gold 250 × 20 mm, Thermo Scientific, Bremen, Germany). The purity of peptide was greater than 98% and was tested by ESI-MS (Esquire 3000, Bruker Daltonics, Bremen, Germany).

### 3.2. Tissue Preparation

Brain cortices were rapidly removed from the male Wistar rats (150–200 g) and frozen immediately on dry ice. Tissues were kept at −80 °C until assayed. Brain was homogenized in an ice-cold 20 mM Tris–HCl buffer, pH 7.5, and diluted 1:6. The obtained homogenate was centrifuged twice at 14,000× *g* for 10 min at 4 °C. The supernatant was divided into small aliquots and kept frozen at −80 °C until usage, as further purification did not change the outcome of the experiments.

### 3.3. Enzyme Separation from Rat Brain Homogenate

Enzymes present in the rat brain homogenate were separated on the ion-exchange column Econo-Pac High Q (resin volume: 5 mL; Bio-Rad, Berkeley, CA, USA). Gradient separation was performed by the HPLC system Prominence LC-20 AD with a Photodiode Array Detector SPD-M20A (Shimadzu, Tokyo, Japan). The sample volume loaded on the column was 1 mL. The mobile phase consisted of 20 mM Tris–HCl buffer, pH 7.5, and a gradient of NaCl from 0 to 500 mM in 60 min was used. The total flow rate was 1 mL/min. One-point-five-milliliter fractions were collected during the separation and stored in −80 °C prior to further experiments.

### 3.4. Enzyme Assay of Homogenized Brain

Enzyme assays were performed in 0.5-mL Eppendorf tubes. Each homogenized brain fraction was diluted 1:5 with 20 mM Tris–HCl buffer, pH 7.5, and the reaction was initiated by the addition of 5 μg of NPNA and continued by mixing at 37 °C for 120 min (or for 300 min for preparing time course metabolic process). Five-microliter aliquots were taken from the incubation mixture, acidified by 30% MeOH with 0.1% FA and analyzed by ESI-MS (Bruker Esquire 3000) equipped with an autosampler (Famos, Thermo Scientific, earlier LC-Packings, Bremen, Germany). MS/MS analysis was performed to confirm the sequence of the released fragments. Quantitation of the enzyme activity was performed by integration of the peak area of formed products on the mass chromatogram. The samples were incubated with and without amastatin, an aminopeptidase inhibitor.

### 3.5. Insulin-Degrading Enzyme (IDE) in Vitro Assays

IDE, His-Tag, rat, recombinant, from *Spodoptera frugiperda*, was purchased from Calbiochem (Milan, Italy), and its activity was verified by carrying out enzymatic digestion of insulin solutions according to the experimental procedure previously reported [29]. Methanol (MeOH), formic acid (FA), Tris–HCl buffer, phosphate buffered saline (PBS) buffer and all chemicals used were purchased from Sigma–Aldrich. ZIPTIP C18 was purchased from Millipore (Milan, Italy). IDE enzymatic assays were carried out by incubating the enzyme solution (0.4 μM) at 37 °C with the peptide solution at the indicated concentration. After purification with ZIPTIP C18, 3 μL of the resulting solution were mixed with 50 μL of water and 50 μL of MeOH and injected into the mass spectrometer at 5 μL/min. ESI-MS experiments were performed by using a Finnigan LCQ DECA XP PLUS ion trap spectrometer operating in the positive ion mode and equipped with an orthogonal ESI source (Thermo Electron Corporation, Madison, WI, USA). Sample solutions were injected into the ion source using nitrogen as the drying gas. The mass spectrometer operated with a capillary voltage of 46 V and capillary a temperature of 250 °C, while the spray voltage was 4.3 kV. MS/MS data were interpreted by using the “Molecular Weight Calculator” software by Matthew Monroe freely available on the web [30].

### 3.6. Surface Plasmon Resonance Measurements

Surface plasmon resonance measurements were carried out on a SensiQ Pioneer instrument, and all reagents used were from Sigma. IDE was immobilized on a COOH5 biosensor chip from ICx Nomadics (Oklahoma City, OK, USA). Briefly, covalent immobilization was obtained by amine coupling of the lysine-free amino groups and terminal amines of the protein, as described elsewhere [31,32]. Particularly, 400 μL of IDE solution at 100 μg/mL in 10 mM acetate buffer, pH 3.8, were used for the immobilization on a previously activated surface having reactive succinimide ester groups obtained by using 1-ethyl-3-(3-dimethylaminopropyl)carbodiimide (EDC) and *N*-hydroxysuccinimide (NHS) solution, freshly prepared ((EDC) = 0.4 M, (NHS) = 0.1 M). NaOH 50 mM was used for regeneration of the surface, while the buffer used for the interaction of IDE with the peptides solutions was obtained by mixing *N*-(2-vsHydroxyethyl)piperazine-*N*'-(2-ethanesulfonic acid) and sodium salt (0.01 M HEPES, 0.15 M NaCl, pH 7.4). The OneStep approach [33] was applied to investigate the interactions between immobilized IDE and NPs and the initial concentrations of the NPSA, NPNA and NPPW peptides solutions were 74, 74 and 150 μM, respectively. The software Qdat was used to fit the experimental curves according to the equations given in [33,34]. Briefly, the signal response (*R*) *versus* time (*t*) is given by:

d*R*/d*t* = *k*_on_ × *C* × *R*_max_ − (*k*_on_ × *C* + *k*_off_) × *R* (1)

where *C* is the analyte concentration, *R*_max_ is the capacity of immobilized ligand and *k*_on_, *k*_off_ are the kinetic constants. For an injection beginning at time t_0_ and ending at *t*_1_, we have:

*c*_1_ = *C* × *k*_on_ + *k*_off_ (2)

For *t*_0_ < *t* < *t*_1_ (association phase):

*R* (*t*) = *C* × *k*_on_ × *R*_max_ × (1 − exp (−*c*_1_ × (*t* − *t*_0_)))/*c*_1_ (3)

For *t* > *t*_1_ (dissociation phase):

*R* (*t*) = *C* × *k*_on_ × *R*_max_ × (1 − exp (−*c*_1_ × (*t*_1_ − *t*_0_)))/*c*_1_ × exp (−*k*_off_ × (*t* − *t*_1_)) (4)

For multi-site kinetics, the total response is given as the sum of response from each individual site. Therefore, for Site 1, *R*_1_ (*t*, *k*_on1_, *k*_off1_, *R*_max1_), and Site 2, *R*_2_ (*t*, *k*_on2_, *k*_off2_, *R*_max2_); total response *R* (*t*) = *R*_1_ + *R*_2_. Fitting is performed using standard Levenberg–Marquardt nonlinear least squares optimization, minimizing the sum-squared error of the difference between the model and the experimental data.

## 4. Conclusions

NPs precursors are inactive and contain multiple copies of various biologically-active sequences. However, the biomolecular mechanisms responsible for the formation of the active peptides are very often obscure. In this work, we have investigated the capability of IDE to degrade various amino acidic sequences “hidden” in the rat and mouse pro-NPFF_A_ precursors, and we have compared the results with those obtained after incubation with rat brain cortex homogenate. Particularly, we have found that IDE is not able to degrade NPNA, while the latter peptide generates several fragments when incubated with rat brain cortex. The results show that the activity of isolated enzymes is not specific, as several proteases cleave NPNA mainly at the carboxyl side of the tryptophan and leucine, converting NPNA peptide to shorter bioactive fragments responsible for the analgesic activity of this rat cryptein. Nevertheless, we have identified one major cleavage site by IDE in a mouse NP of the same amino acidic length, that is NPSA. These results demonstrate that IDE is capable of the degradation of substrates that are as short as 15 amino acidic residues, but is also sequence specific. However, as NPNA is degraded in rat brain cortex, IDE does not seem to be involved in this process. Indeed, although IDE’s ability to degrade peptide is not highly substrate specific, this enzyme does not degrade all peptides [35], and it is ubiquitously present in the nervous tissue. For this reason, we decided to explore the possibility that IDE is indeed involved in the proteolysis of NPFF fragments, in particular cryptic sequences, as they became an emerging source of potential therapeutics. As the results of our work show, there are several enzymes involved in the cleavage of NPNA. The key point of our work was therefore to reveal possible fragments generated in the brain. This is important for two reasons. The first is purely pharmacological, because NPNA analogs can be used as a potential therapy in drug dependence [12]. Another purpose was to investigate the potential NPNA endogenous fragments that may exist in the brain. Therefore, this work did not aim at the isolation and identification of such sequences, because this requires specific antibodies and affinity chromatography. In turn, antibodies can be raised only when the appropriate fragments have been recognized.

On the other hand, we have found that the most abundant peptide fragment (PQRFGRNAWGPWSKEQLSPQ) that is generated when the elongated peptide sequence NPPW 1–25 is incubated with IDE is also produced in a high amount when the same peptide is incubated in rat brain cortex. The latter result indicates that IDE is very likely to be involved in the degradation of NPPW 1–25 *in vivo*. This finding tends to prove the recently proposed hypothesis according to which IDE has many different roles *in vivo* [24], which have still to be unveiled.

Finally, the *K*_D_ constants for the interaction between IDE and the three investigated NPs have been obtained by SPR analysis. The results show that the SPR curve for NPNA, the only NP among the scrutinized peptides that is not degraded by IDE, can be better fitted by using the one binding site model. On the contrary, the SPR curves recorded for the interaction between IDE and the other two peptides, NPSA and NPPW, require the two binding sites model to be fitted. Such results could be explained by considering that, in order to act as a degrading enzyme, IDE has to bind the substrate through two different binding sites. In the cases explored above, it is likely that the second binding site of IDE is sitting between the last amino-acids of the NPPW peptide (REFW) of the sequence, as they do not belong to the NPNA sequence and constitute the target of IDE. However, such speculation needs to be further verified by studying the interaction of IDE with many other biomolecules.

## Supplementary Materials

Supplementary Figures can be found at http://www.mdpi.com/1422-0067/15/9/16787/s1.

## Supplementary Files

Supplementary File 1
