# Supplementary material for: Metabolism of Cryptic Peptides Derived from Neuropeptide FF Precursors: The Involvement of Insulin-Degrading Enzyme
Source: Int J Mol Sci. 2014 Sep 22;15(9):16787–99. doi: 10.3390/ijms150916787 (PMC4200852; doi:10.3390/ijms150916787)

Supplementary Information

Figure S1. Time dependent data: (**A**) Time dependent incubation of NPNA with enzymes from fraction number 23. The main product is created in the highest amount after 4 h, fragment 1–11 [NAWGPWSKEQL]; (**B**) Time dependent incubation of NPNA with enzymes from fraction number 26. The main product is fragment 7–11 [SKEQL]; (**C**) Time dependent incubation of NPNA with enzymes from fraction number 35. The only identified product is fragment 4–15 [GPWSKEQLSPQA].

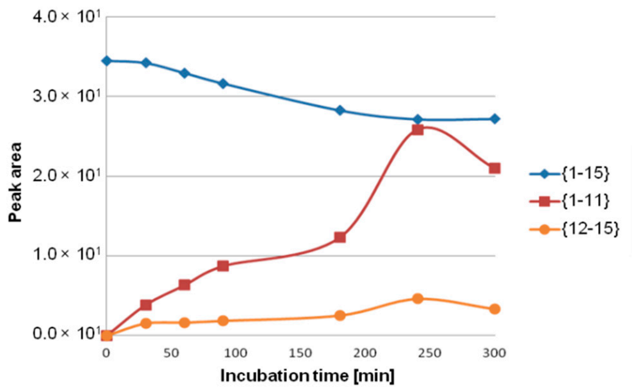

(**A**)

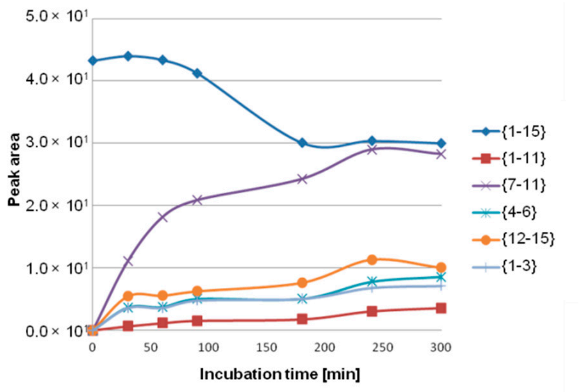

(**B**)

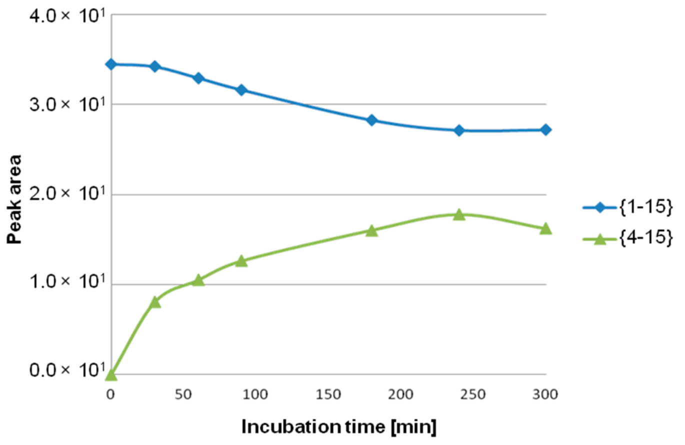

(**C**)

Figure S2. MS/MS spectrum for the peak at *m*/*z* 693.2, corresponding to peptide SAWGSW.

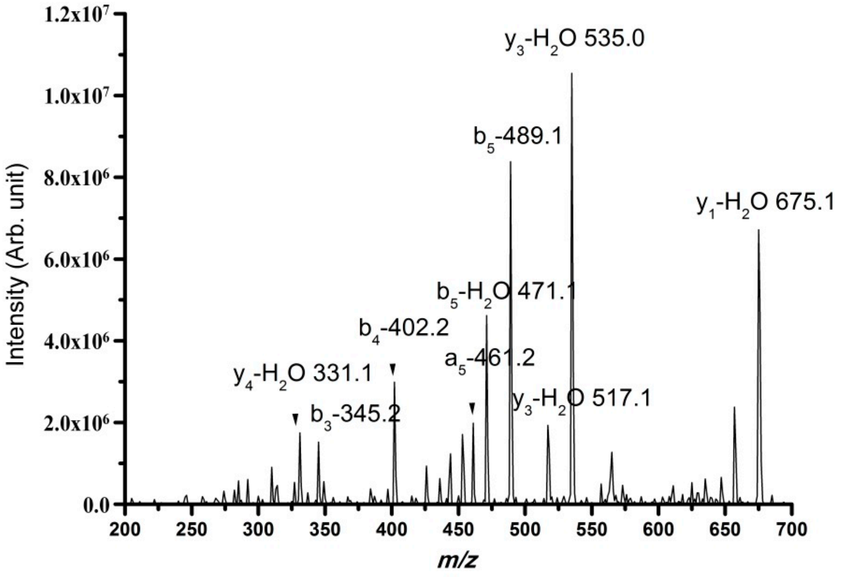

Figure S3. MS/MS spectrum for the peak at *m*/*z* 1442.7, assigned to fragment WGPWSKEQLSPQ.

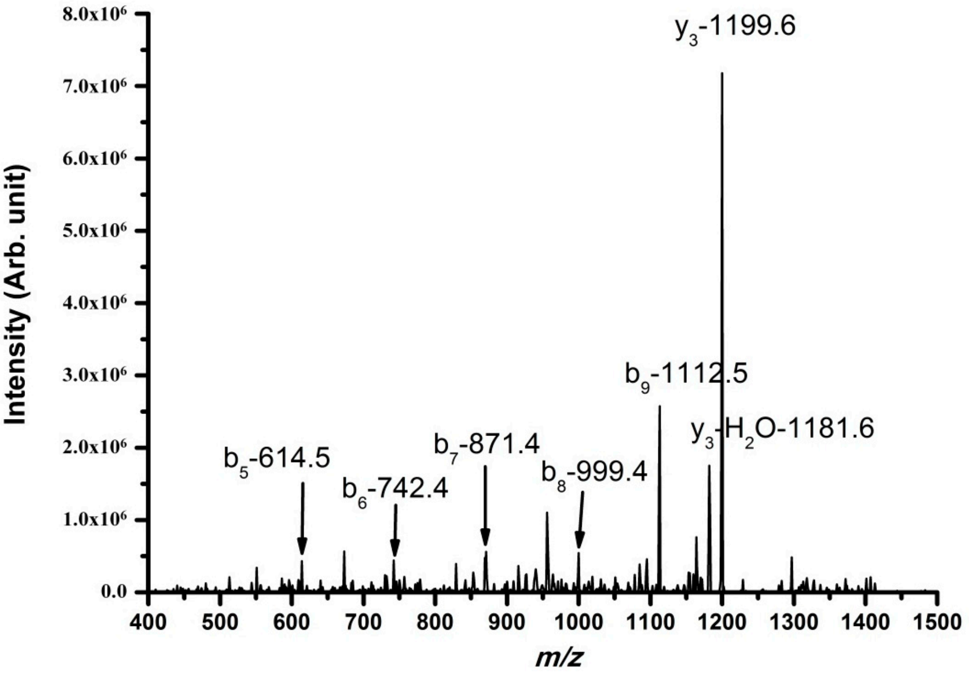

Supplement: Supplementary File 1 [file ijms-15-16787-s001.pdf]
